# Location, speciation, and quantification of carbon in silica phytoliths using synchrotron scanning transmission X-ray microspectroscopy

**DOI:** 10.1371/journal.pone.0302009

**Published:** 2024-04-15

**Authors:** Djanira R. Negrao, Julio C. Cezar, Fabiano E. Montoro, Jian Wang, Charles W. Rice, Carlos E. Driemeier

**Affiliations:** 1 Brazilian Biorenewables National Laboratory (LNBR), Brazilian Center for Research in Energy and Materials (CNPEM), Campinas, SP, Brazil; 2 Department of Agronomy, Kansas State University, Manhattan, KS, United States of America; 3 Brazilian Synchrotron Light Laboratory (LNLS), Brazilian Center for Research in Energy and Materials (CNPEM), Campinas, SP, Brazil; 4 Brazilian Nanotechnology National Laboratory (LNNano), Brazilian Center for Research in Energy and Materials (CNPEM), Campinas, SP, Brazil; 5 Canadian Light Source (CLS), Saskatoon, SK, Canada; Universite Libre de Bruxelles, BELGIUM

## Abstract

Phytoliths of biogenic silica play a vital role in the silicon biogeochemical cycle and occlude a fraction of organic carbon. The location, chemical speciation, and quantification of this carbon within phytoliths have remained elusive due to limited direct experimental evidence. In this work, phytoliths (bilobate morphotype) from the sugarcane stalk epidermis are sectioned with a focused ion beam to produce lamellas (≈10 × 10 μm^2^ size, <500 nm thickness) and probed by synchrotron scanning transmission X-ray microspectroscopy (≈100–200 nm pixel size; energies near the silicon and carbon K-absorption edges). Analysis of the spectral image stacks reveals the complementarity of the silica and carbon spatial distributions, with carbon found at the borders of the lamellas, in islands within the silica, and dispersed in extended regions that can be described as a mixed silica-carbonaceous matrix. Carbon spectra are assigned mainly to lignin-like compounds as well as to proteins. Carbon contents of 3–14 wt.% are estimated from the spectral maps of four distinct phytolith lamellas. The results provide unprecedented spatial and chemical information on the carbon in phytoliths obtained without interference from wet-chemical digestion.

## 1. Introduction

A variety of plant species form micrometric structures of biogenic silica called phytoliths. Phytoliths are formed by the uptake of monosilicic acid from the soil through the roots, followed by the deposition of hydrated amorphous silica in cell walls, cell lumens, and intercellular spaces of roots, leaves, stems, and hulls [1–5]. Phytoliths play structural roles in plants, contributing to strength and defense against abiotic and biotic stresses (*e*.*g*., herbivory and fungal attacks) [6–8]. In addition, phytoliths have a fundamental role in the biogeochemical silicon cycle [5, 9] and take part in biomass biorefining into bio-based renewable products [10–12]. Phytoliths are abundant in grasses (*Poaceae*), which underscores the importance of phytoliths for grasslands and major global crops such as corn, wheat, rice, and sugarcane [9, 13–15].

Carbon species are found trapped within the silica phytoliths, the so-called phytolith occluded carbon (PhyOC). Because of phytolith abundance, characteristic morphology, and durability in terrestrial ecosystems, PhyOC has been used for radiocarbon dating in archeology, paleontology, and related disciplines [16–19] and has attracted attention as a potential path for carbon sequestration [16, 20–23]. In this regard, fluxes of PhyOC in crops like sugarcane [14, 24], wheat [25], rice [26–29], and palm [30] as well as in grasslands [31], and forests [32, 33] have been documented. However, the potential of PhyOC for radiocarbon dating and carbon sequestration has sparked controversy due to the potential inclusion of carbon sourced from soil and challenges in determining the content and durability of PhyOC across different types of phytoliths [16, 21–23, 34–37].

Due to its importance and remaining uncertainties, measuring undisturbed carbon in phytoliths has been a target for research. Seminal work by Harrison [38] used wet chemistry to access the organic compounds within silica phytoliths. Amino acids were detected, indicating the presence of protein within the biogenic silica. Later, more direct analysis of carbon in phytoliths used nanoscale secondary ion mass spectrometry (nanoSIMS) [35] and Raman spectromicroscopy [39]. Both studies revealed carbon dispersed throughout the silica. However, while the nanoSIMS study detected N/C ratios consistent with protein within the silica [35], the Raman study highlighted the variability observed among the phytoliths and the observation of spectral features consistent with carbohydrates, lignins, and other types of organic matter within the phytoliths [39]. Variability in spectral features was also used to discriminate distinct types of sorghum phytoliths and to show how the wet chemical method used to isolate the phytolith impacts the measured organic matter [40]. In addition, Dynamic Nuclear Polarization Nuclear Magnetic Resonance (DNP NMR) was used by Masion et al. [41] to evaluate carbon in phytoliths, which showed peptides and carbohydrates as predominant compounds. However, all these previous studies relied on wet chemical digestion to isolate phytoliths before analysis, which interferes with the organic matter detected so far.

Here we report a method free of wet chemical digestion to determine the spatial distribution, chemical speciation, and quantification of carbon within silica phytoliths. Sugarcane stalk epidermis containing bilobate phytoliths are sectioned by a focused ion beam coupled to scanning electron microscopy (FIB-SEM) to obtain <500 nm thick phytolith lamellas. These lamellas, which are transparent to X-rays, are mapped with 100–200 nm pixel size by synchrotron scanning transmission X-ray microspectroscopy (STXM) with spectral analysis of the silicon and carbon K-absorption edges. The method is employed to section and analyze four phytoliths, revealing spectral signatures and spatial maps of the silicon and carbon species in the phytoliths.

## 2. Materials and methods

### 2.1 Preparation of phytolith lamellas

Epidermal regions of sugarcane stalk internodes were isolated from a 1-year-old sugarcane plant (CTC-4 variety) harvested in February 2021 in the LNBR/CNPEM garden. Pieces of 0.5 mm × 0.5 mm × 2 mm were preserved in a formaldehyde-glutaraldehyde buffer (3 days/4°C), dehydrated in a series of acetone/water solutions (30, 50, 70, and 90%), and dried with critical point (CPD 300, Balzec, Lichtenstein) using CO_2_ for gas exchange. These pieces (which contain undisturbed silica phytoliths) were mounted onto aluminum stubs with carbon tape and coated with a <10 nm gold nanolayer to minimize charging during the preparation of phytolith lamellas and imaging with FIB-SEM.

FIB-SEM with a gallium ion beam was employed to obtain <500 nm thick lamellas directly from four distinct bilobate phytoliths sectioned in the sagittal plane. Such bilobate phytoliths, a type of grass silica short-cell phytolith, populate the surface of the sugarcane stalk internode. They are aligned to plant fibers and have lengths of about 21–25 μm and lobe widths of about 11–15 μm [11]. Before sectioning, a strip of platinum (approximately 15 μm length × 2 μm width × 1 μm height) was deposited (beam current of 0.50 nA at 30 kV) to prevent surface damage by the ion beam. Then, two trenches (approximately 25 μm length × 15 μm width × 15 μm height) were opened adjacent to the platinum strip by milling with FIB (beam current of 30 nA and cleaning with 7 nA, both at 30 kV). This step yields lamellas (approximately 15 μm length × 15 μm height × 2 μm thickness) still attached to the epidermal tissue at the edges and the bottom.

**Fig 1:**
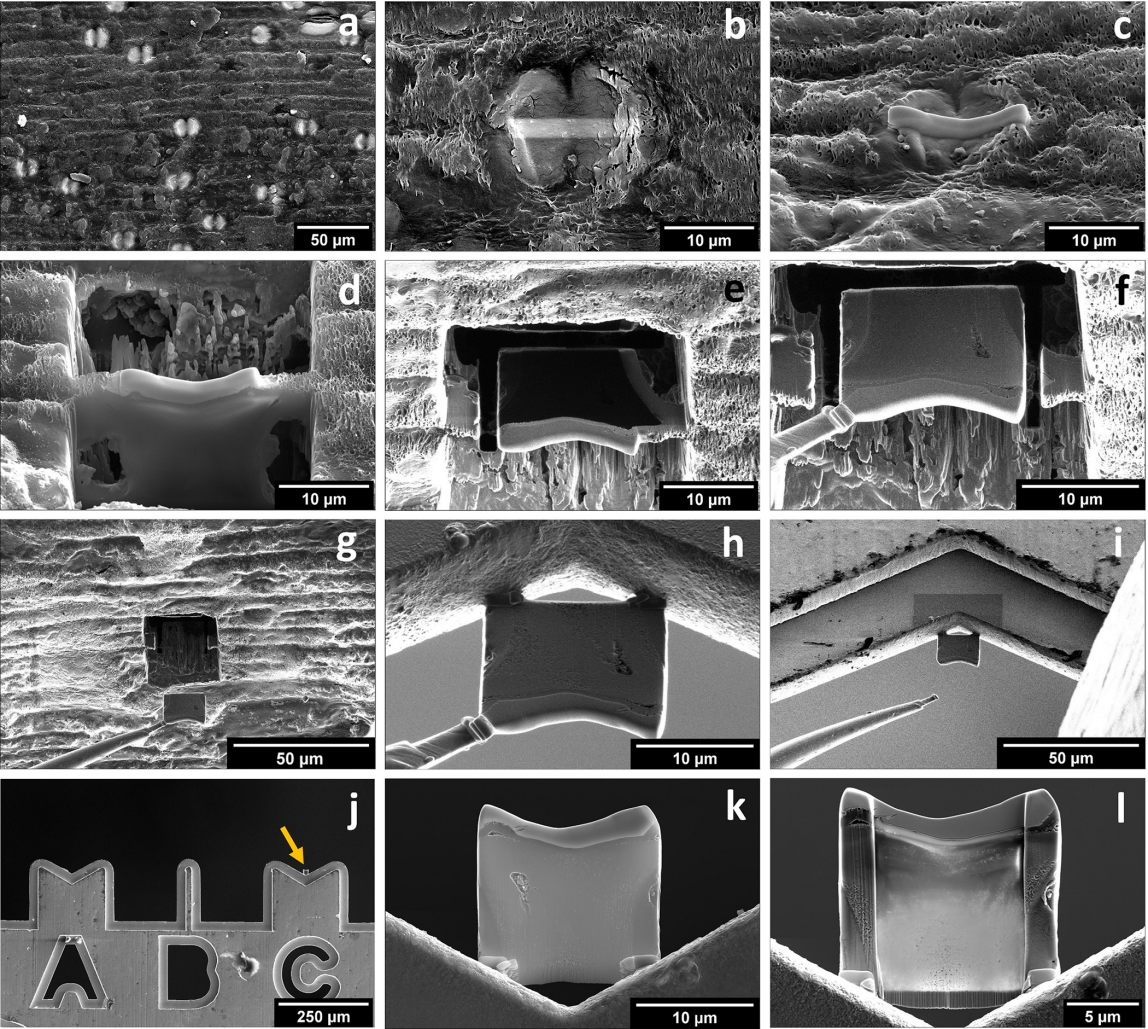
Scanning electron micrographs of the preparation steps of phytolith lamellas. (a) bilobate phytoliths at the surface of the sugarcane stalk. (b-c) Platinum cover strip deposited along the longitudinal axis of a phytolith. (d-e) Trenches carved by the focused ion beam to form the phytolith lamella. (f-g) Fixation of the micromanipulator to the platinum strip and removal of the lamella from the plant tissue. (h-i) Fixation of the lamella to a copper lift-out grid (j). The arrow points to the lamella in (j). Detail of the phytolith lamella fixed to the lift-out grid before (k) and after (l) thinning (<500 nm) the central region of the lamella under the focused ion beam.

The transfer of self-sustained lamellas to a copper grid was performed with the beam at 30 kV through the following five steps. *i*) cutting the right edge and the bottom of the lamella from the plant tissue (beam current of 3 nA); *ii*) welding the microprobe manipulator with platinum at the upper left lamella corner (beam current of 50 nA), *iii*) cutting the remaining left edge attached to the plant tissue (beam current of 3 nA), *iv*) transferring the lamella to the FIB lift-out copper grid (Ted Pella®), and *v*) fixing the lamella to the copper grid by welding platinum at its left and right corners (beam current of 10 nA). The final step in preparing the lamella was to thin the central region using currents ranging from 500 pA to 100 pA at 30 kV. The final thickness achieved was below 500 nm. The steps for the preparation of phytolith lamellas can be visualized in **Fig 1**.

### 2.2 Synchrotron scanning transmission X-ray microspectroscopy (SXTM)

Phytolith lamellas were fixed to the sample grids and shipped to the Canadian Light Source (CLS) where the experiments were conducted on the Scanning Transmission X-ray Microscope (STXM) in the soft X-ray spectromicroscopy beamline (beamline 10ID-1). The microscope chamber was pumped down to 100 mTorr and then backfilled with ≈0.2 atm of helium gas. In STXM, the monochromatic X-ray beam was focused by a Fresnel zone plate to a typical spot size of about 50 nm and landed on the surface of the lamella. The detector recorded the transmitted X-ray intensity. The sample was scanned in the (x,y) plane to generate transmission images with slightly larger pixel sizes of 107–190 nm (varying for different image stacks) than the beam spot size to be more efficient for data acquisition and reduce radiation damage. Image stacks were acquired by scanning the photon energies in the ranges 1835–1895 eV and 280–319 eV for the silicon K edge and carbon K edge spectra, respectively. The spectral ranges were shortened respectively to 1840–1880 eV and 280–310 eV for the presentation of the analyzed data.

### 2.3 Spectral data analysis

The STXM data were processed in the Mantis software package [42]. All the STXM image stack spectra were converted to optical densities (OD) using the spectra recorded outside the sample region as the intensity reference (I_0_). Each image stack was aligned by cropping a selected area within the thin section and manipulating image displacements using Gaussian and linear regression filters. Corrupted images were deleted before spectra processing.

Rectangular regions of interest within the phytolith lamellas were selected for spectral analysis. For each lamella, the regions selected for each energy range (1840–1880 eV and 280–310 eV) were tentatively identical. Nevertheless, ensuring perfectly equal areas was not feasible due to the independent acquisition of image stacks at different energies for carbon and silicon K-edges.

The image stacks were analyzed by Principal Component Analysis (PCA). The number of significant components was chosen as one for the 1840–1880 eV stacks and two for the 280–310 eV stacks, as justified in the results section. Target spectra were constructed from the eigenspectra obtained from PCA. For each 1840–1880 eV stack, the PC1 eigenspectrum was defined as the target spectrum. For each 280–310 eV stack, two target spectra were constructed as linear combinations of the PC1 and PC2 eigenspectra.

After constructing the target spectra, spectral weight maps were generated using the fact that each measured image stack *D*_*np*_ can be approximated by a set of target spectra *μ*_*ns*_ and spectral weight maps *t*_*sp*_, where the indices *n*, *p*, and *s* represent the energy, pixel, and spectral component, respectively [43]. For each 1840–1880 eV image stack, the corresponding PC1 eigenspectrum was used as the single target spectrum to obtain the silicon weight map. Meanwhile, for each 280–310 eV image stack, the pair of target spectra was used to obtain the corresponding pair of weight maps.

## 3. Results

### 3.1. Visualization of the regions selected for spectral analysis

**Fig 2:**
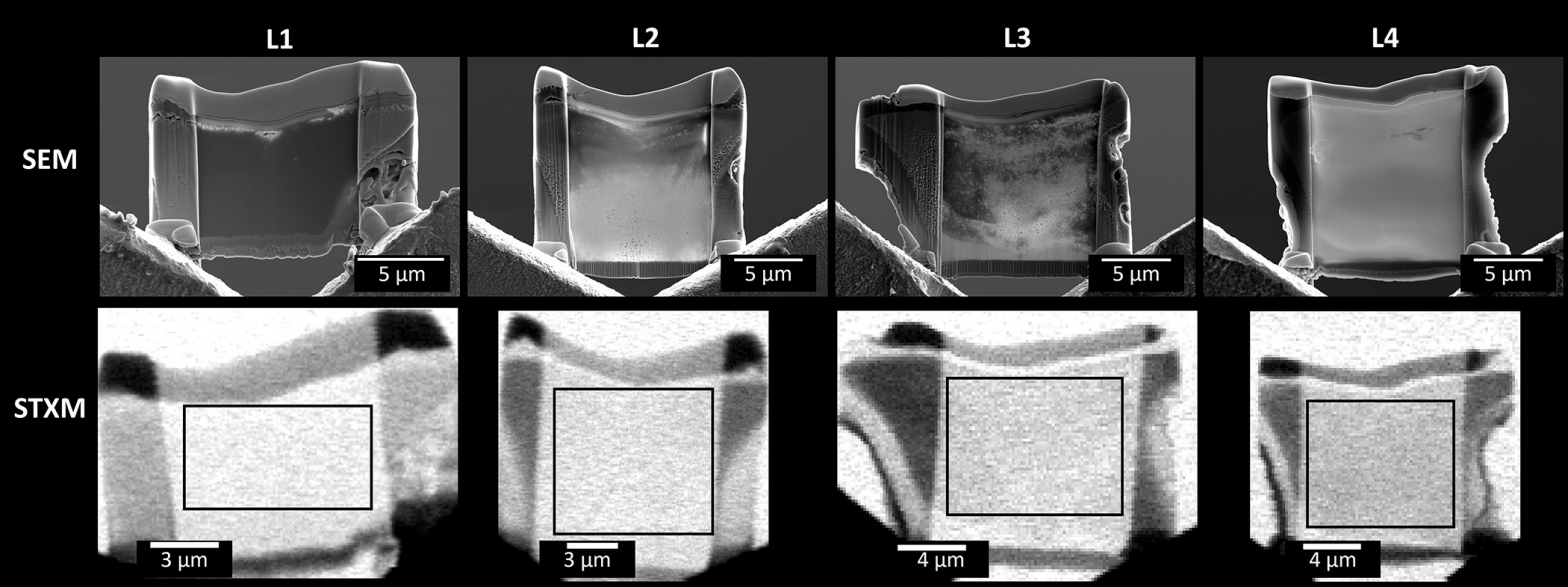
Panoramic images of the phytolith lamellas. The images were obtained by Scanning Electron Microscopy (SEM, secondary electrons, top) and Scanning Transmission X-ray Microspectroscopy (STXM, photon energy 285.0 eV, bottom) from the four mounted phytolith lamellas (L1–L4). The rectangular regions selected for spectral analysis are approximately indicated in the STXM images.

The four mounted phytolith lamellas (L1–L4) fixed onto the lift-out grids were imaged by SEM (secondary electrons mode) before the STXM data acquisition. **Fig 2** shows both SEM and STXM panoramic images of L1–L4, where the fields of view include the lamellar regions thinned for X-ray transmission, the surrounding plant tissues and sample holder that serve as mechanical support, and external regions that are used as the X-ray transmission reference (I_0_). The aim of selecting regions for spectral analysis was to ensure (i) the rectangle areas are entirely within the thinned lamellas and (ii) the regions used for the two energy ranges (1840–1880 eV and 280–310 eV) are approximately aligned. In addition, the selected regions are positioned mainly within the phytoliths, although regions external to the phytoliths (i.e. plant tissue) may appear near the border of the analyzed rectangles.

### 3.2. Eigenspectra and spectral maps

**Fig 3:**
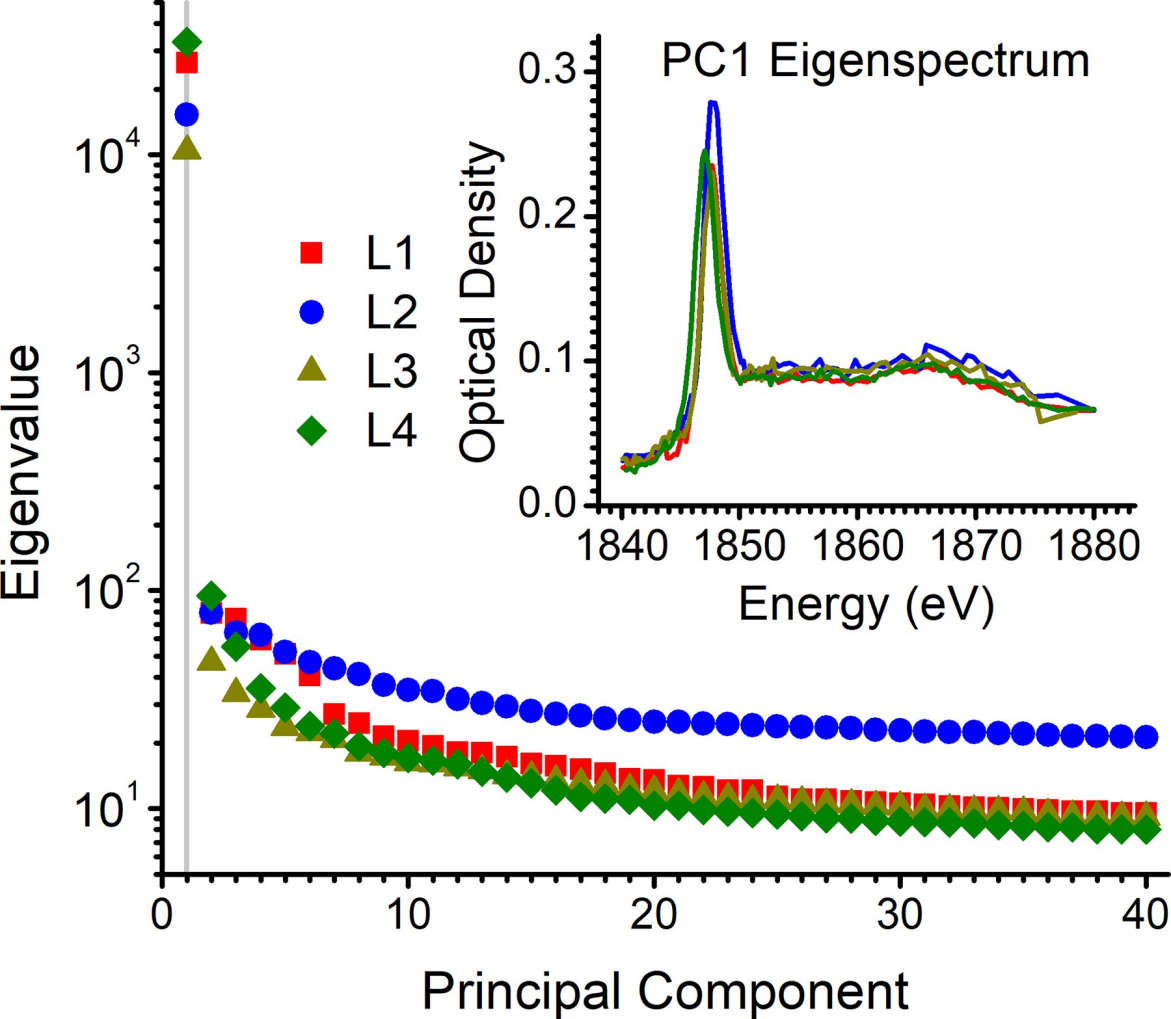
Principal component analysis of the image stacks in the 1840–1880 eV energy range. The scree plot indicates the significance is limited to PC1 for each one of the four phytolith lamellas (L1–L4). The inset shows the corresponding PC1 eigenspectra, which are consistent with opaline silica.

Spectral analysis by PCA allows the decomposition of the STXM image stacks into eigenspectra and spectra maps. After PCA, the number of principal components (PCs) that are significant enough to be carried out for further analysis needs to be judged. The scree plot of the PCA performed for the 1840–1880 eV image stacks (**Fig 3**) shows that the eigenvalues of PC1 are two orders of magnitude higher than PC2, followed by smooth decay of the eigenvalues for the next PCs. This pattern indicates that PC1 alone carries the significance of the image stacks, a pattern consistent across L1–L4. The PC1 eigenspectra at the silicon K edge (**Fig 3** inset) are almost identical for L1–L4 and the spectral profiles are consistent with either amorphous or opaline silica [44]. Knowing that phytoliths are made of opal [45, 46], these results demonstrate that a single type of silicon species (opal) is present in the analyzed regions of L1–L4, with variations attributed only to the amount (thickness) of silica at different positions (pixels) of the lamellas.

The carbon K edge energy range (280–310 eV) is analyzed to complement the spectral information obtained from silicon. The scree plot of the PCA performed for the 280–310 eV image stacks (**S1 Fig**) shows that the eigenvalues of PC1 and PC2 are much higher than the eigenvalues of PC3 and beyond. This pattern is consistent across L1–L4 and indicates that PC1 and PC2 are the significant components to be used for further analysis. The PC1 and PC2 eigenspectra in the 280–310 eV range (**S1 Fig**) present variations across L1–L4, and so do the associated PC1 and PC2 eigenimages (**S2 Fig**). Recalling that the eigenspectra are abstract constructs based on statistical patterns [43], linear combinations of the eigenspectra are built as: (i) linear (*i*.*e*., non-resonant) intensity, which is constructed as the linear combination of eigenspectra that best approximates a straight line; and (ii) carbon K edge spectrum, which is constructed as the linear combination that sets the pre-edge region (~280 eV) close to zero. These spectral combinations are named *target spectra* (**S1 Fig**) and are used as such in the Mantis software [42].

**Fig 4:**
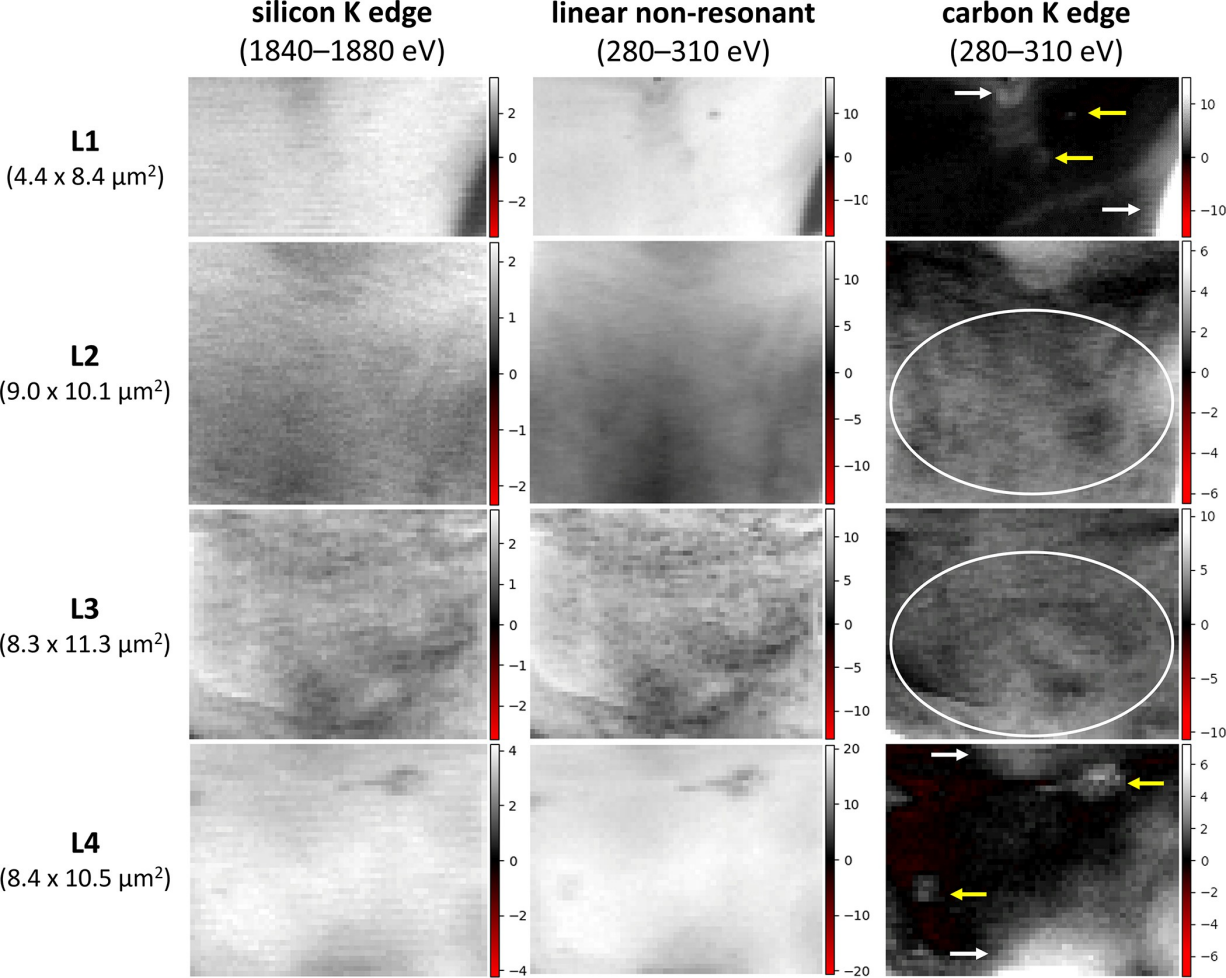
Spectral weight maps obtained for the phytolith lamellas L1–L4. (left) Maps of the silicon K edge PC1 eigenspectrum in the 1840–1880 eV stacks. Maps of the linear (center) and carbon K edge (right) components in the 280–310 eV stacks. In the carbon K edge maps, carbon is concentrated near the lamella borders (white arrows), as gray islands (yellow arrows), and as areas of carbon dispersed in the silica matrix (ellipses).

After constructing the target spectra for the 1840–1880 eV (**Fig 3**) and 280–310 eV (**S1 Fig**) image stacks, the spectral maps of the phytolith lamellas L1–L4 are obtained (**Fig 4**). The maps show the spatial distribution of the spectral components, where black represents zero and red represents negative (unphysical) spectral weight. The shades of gray from black to white represent increasing spectral weight, which is proportional to the amount (thickness) of the component at a particular spatial position (pixel).

The key point observed between the spectral weight maps is the similarity between the maps obtained from the silicon K edge eigenspectrum at 1840–1880 eV (**Fig 4**, left column) and the linear spectral component at 280–310 eV (**Fig 4**, center column). This similarity is consistent across L1–L4. This result demonstrates that the linear component in the 280–310 eV energy range is mainly due to the non-resonant X-ray absorption by the silica matrix. Although this interpretation may be straightforward, the unique aspect is that these similar maps were generated from independent measurements and analysis (at 1840–1880 eV and 280–310 eV) of each lamella, which underscores the strength and reliability of the experimental and data analysis methodologies employed in this work.

A second observation is given to the complementarity of the carbon K edge spectral weight maps (**Fig 4**, right column) compared to the linear component in the same spectral region (**Fig 4**, center column) or the silicon maps (**Fig 4**, left column). This complementarity (akin to a negative image) probably arises from the competition between silica and carbon compounds to occupy the space within the lamellas. Consequently, an area (pixel) rich in carbon tends to have less silicon, and vice versa. This complementarity reinforces confidence in accurately localizing the carbon within phytolith.

Three types of carbon location can be discriminated at the carbon maps (**Fig 4**, right column): (i) Carbon concentration is notably observed near the borders of the spectral maps L1–L4. However, the primary concern is the uncertainty whether this border carbon exists inside or outside of the silica matrix. (ii) Carbon in localized spots is visible as gray islands. Such islands are recognized only in L1 and L4 (see arrows in **Fig 4**) and they likely represent the prototypical type of carbon that is completely occluded by the silica matrix. (iii) Carbon dispersed in the silica matrix is visible in all lamellas (L1–L4), with greater prominence in L2 and L3. This form of carbon appears as dispersed clouds with varying shades of gray, making it challenging to definitely outline each carbon region. It is worth mentioning that discerning these three types of carbon is only possible because of the high spatial resolution (pixel size ≈100–200 nm) of the experimental approach used in this study.

### 3.3. Carbon chemical speciation

**Fig 5:**
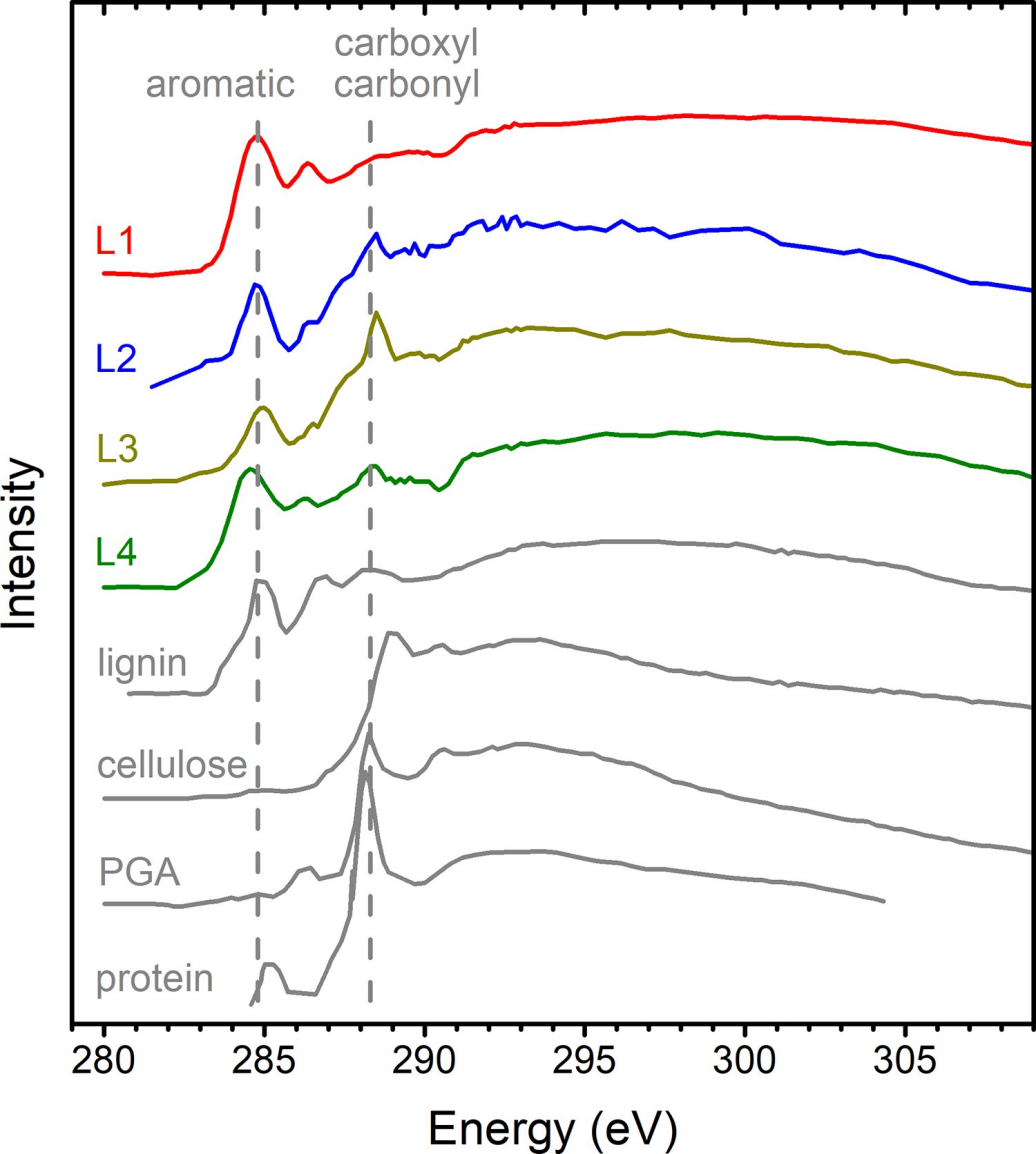
X-ray absorption spectra in the carbon K edge. (Colored) Target spectra were obtained from the linear combination of the eigenspectra of the image stacks measured in the phytolith lamellas (L1–L4) of the present study. (Gray) Reference spectra of protein reported by Zubavichus et al. [48] and of plant cell wall biopolymers lignin, cellulose, and polygalacturonic acid (PGA) reported by Karunakaran et al. [47] after shifting the energy axis (–0.2 eV). Approximate positions of peaks from aromatic (aryl), carboxyl (O = COH), and protein carbonyl (O = CN) groups are marked.

The carbon K edge target spectra (**S1 Fig**) are reproduced in **Fig 5** to inform the carbon chemical speciation. The carbon spectra exhibit the most prominent peak at ≈285.0 eV, with a second peak observed at ≈288.5 eV. The peak at ≈285.0 eV is assigned to carbon π* resonance in the aromatic (aryl) group, while the peak at ≈288.5 eV is consistent with π* resonances in carboxyl (O = COH) or in carbonyl of proteins (O = CN) [47, 48]. Both peaks are evident across L1–L4; however, the peak at ≈288.5 eV is notably broader and less defined in L1. Moreover, the relative intensities of the aromatic and carboxyl/carbonyl peaks are variable, and higher aromatic intensity is related to lower carboxyl/carbonyl, and vice versa.

Comparison with previous X-ray absorption studies of proteins [48] and plant cell wall biopolymers [47] reveals similarities with the carbon K edge spectra obtained in this work (**Fig 5**). The reference spectrum of cellulose displays characteristics that do not align with the observations of the carbon K edge spectra from the phytolith lamellas. The same inconsistency can be affirmed for the other cell wall carbohydrates, such as xylans and β-glucans [47]. Therefore, the observed carbon signatures in these sugarcane phytoliths cannot be attributed to these cell wall carbohydrates. On the other hand, the reference spectra of protein, lignin, and polygalucturonic acid (PGA) present features consistent with the observations in phytolith lamellas. Lignin, protein, and PGA present prominent peaks at ≈288.5 eV, while lignin and protein present prominent peaks of aromatic groups at ≈285.0 eV. Noteworthy, the reference spectra of **Fig 5** were not obtained from sugarcane. The protein was obtained from ovalbumin, lignin from hydrolytic lignin from a commercial supplier, and PGA from citrus fruit [47, 48]. Therefore, due to their different origins, some disagreement with the reference spectra is unavoidable. Taking everything into account, determining the relative contribution of lignin-like aromatic compounds, protein, and PGA to the ≈288.5 eV peak is somewhat uncertain. However, the high intensity of the aromatic peaks at ≈285.0 eV strongly suggests that lignin-like compounds constitute a significant portion of the carbon within phytoliths. The spectral attribution is further developed in the Discussion section.

### 3.4. Raw spectra at selected regions of interest

**Fig 6:**
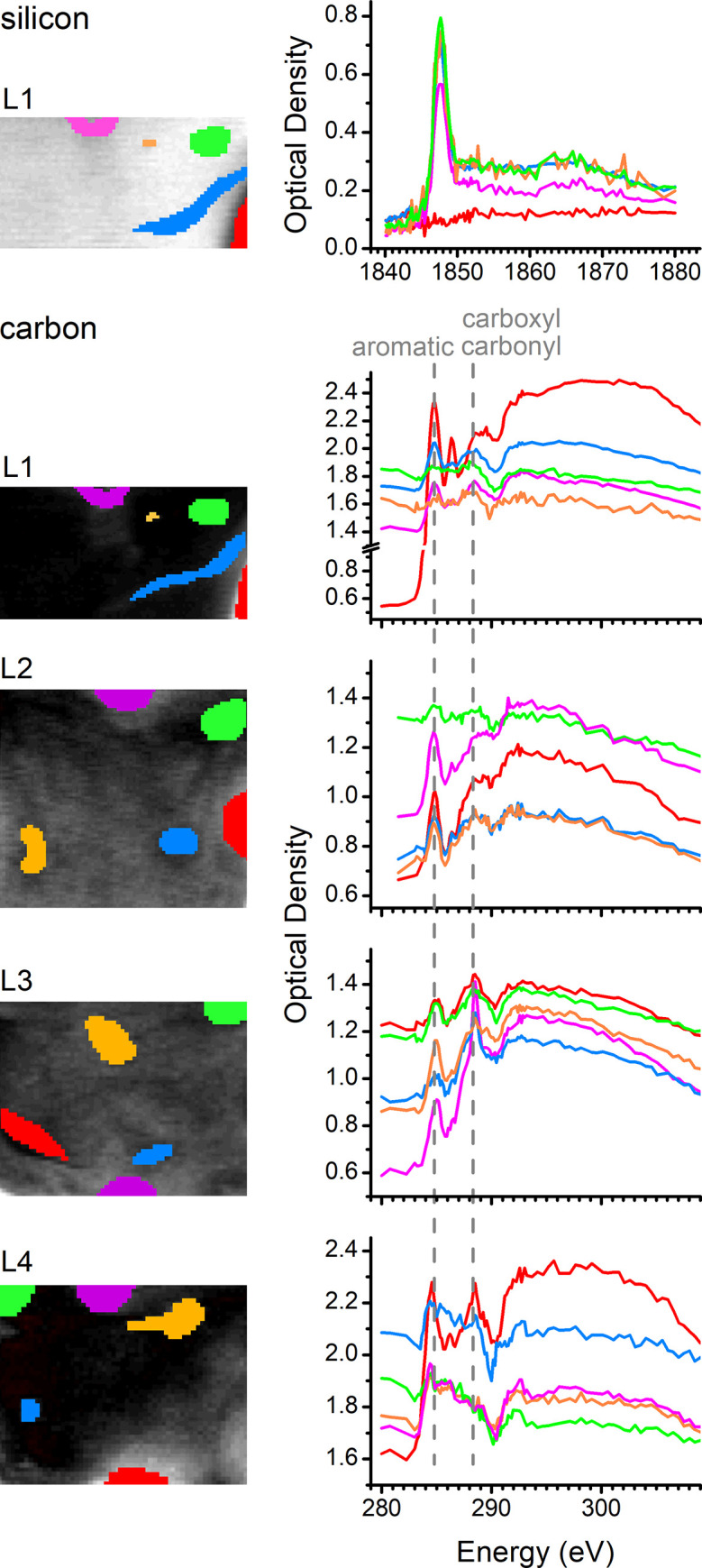
Selected regions of interest (ROIs) and their measured spectra. At the top, selected ROIs are presented for the silicon K edge energy range (1840–1880 eV), having L1 as an example of phytolith lamella. Selected ROIs are also presented for the four phytolith lamellas (L1–L4) in the carbon K edge energy range (280–310 eV).

The spectral maps (**Fig 4**) showed where phytolith carbon is located, while the carbon spectra (**Fig 5**) evidenced carbon spectral signatures assignable mainly to lignin, possibly mixed with protein and PGA. Nevertheless, these results arise from the statistical analysis of the 280–310 eV image stacks. To verify to what extent these findings are supported by raw spectral data, regions of interest (ROIs) are selected from the maps and displayed alongside their corresponding measured spectra (**Fig 6**).

The silicon K edge spectra of L1 (top of **Fig 6**) show the same type of spectral profile of the PC1 eigenspectrum obtained from the 1840–1880 eV stacks (**Fig 3**). The important exception here is the region at the bottom right corner of L1, which lacks the silicon signal. This region will be henceforth used as a reference region with no silica.

The raw spectra in the carbon K edge energy range (280–310 eV) at the selected ROIs (**Fig 6**) show a diversity of background and peak intensities that are broadly consistent with the maps of **Fig 4** and the spectra of **Fig 5**. It is worth highlighting the very intense carbon edge at the bottom right corner of L1, which confirms that the region of zero silica is rich in carbon with a spectrum similar to lignin (**Fig 6**). This ROI in L1 is henceforth considered 100% lignin for use as a reference in the dataset.

The aromatic peak at ≈285.0 eV is the most remarkable feature in most of the spectra of **Fig 6**, confirming that lignin-like compounds are dominant carbon species within the phytolith lamellas. On the other hand, the ≈288.5 eV peak is observed with variations in width, consistent with different carbon species (lignin, protein, and PGA) contributing to this peak in different regions of phytolith lamellas. This difference is more remarkable in the bottom ROIs of L3, where the ≈288.5 eV peaks are notably sharper than elsewhere.

### 3.5. Carbon quantification

**Fig 7:**
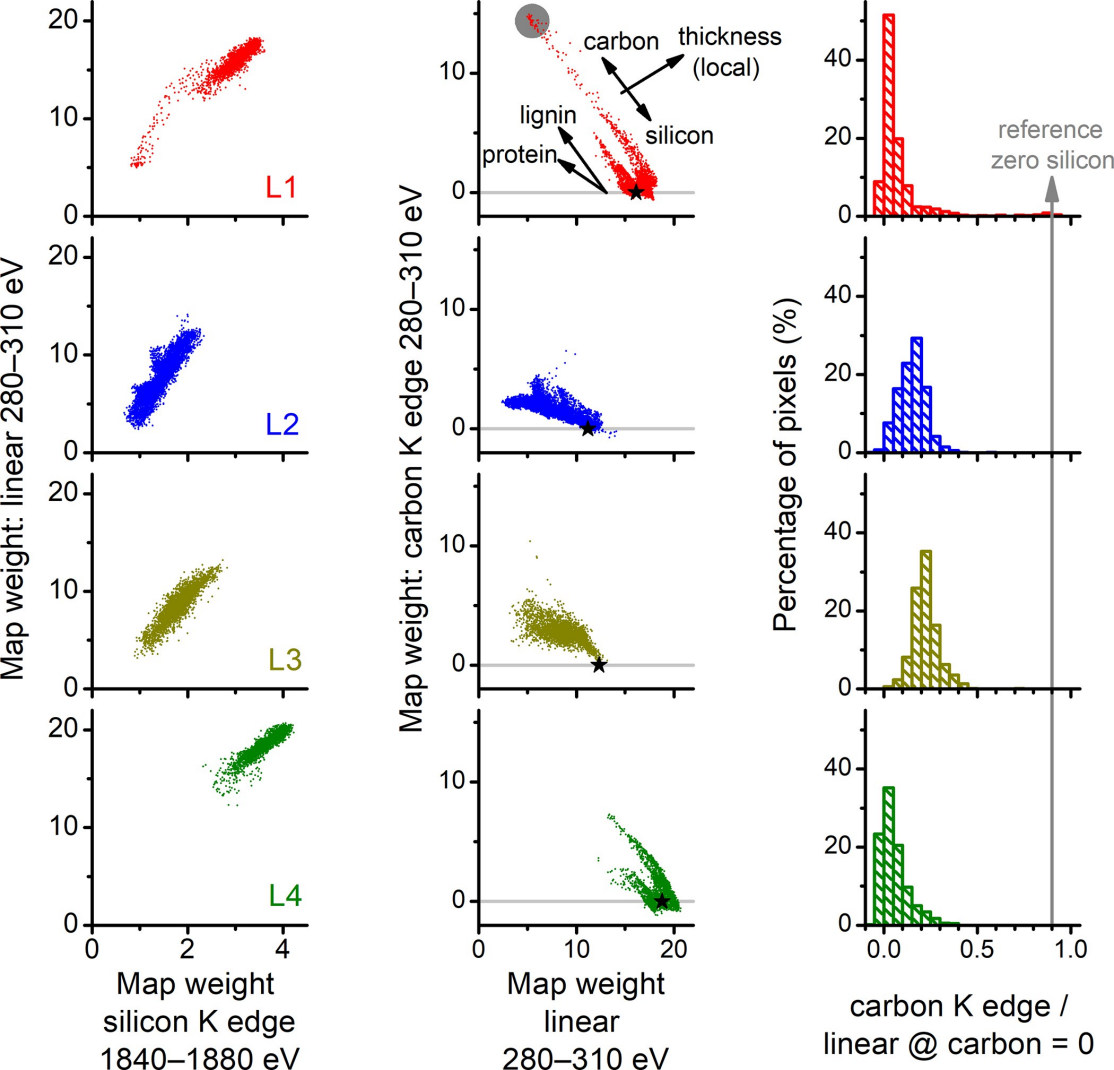
Analysis of the spectral weight maps for the L1–L4 phytolith lamellas. (left column) Scatter plots of weights obtained from the linear component in the 280–310 eV stacks versus the silicon K edge in the 1840–1880 eV stacks, evidencing proportionality. (center column) Scatter plots of weights obtained from the carbon K edge and the linear components in the 280–310 eV stacks, evidencing negative correlations. In the center top graph, the datapoints are distributed across diagonal streak lines, whose interpretation is illustrated by the arrows. Weights in the x and y axes tend to increase with local lamella thickness. The complementarity of carbon and silicon tends to disperse the datapoints across streaks of negative slope that scale with carbon density, which is, for example, higher for lignin than protein. (right column) Histograms of carbon signals are calculated by dividing the carbon K edge weights (pixel value in the y-axis in the center column graphs) by the mean linear weight of each lamella where the carbon K edge weight is approximately zero (x value of the stars in the center column graphs). The reference of zero silicon is marked as a vertical line (right column) and a gray circle (top graph of the central column).

The quantification of carbon in phytoliths involves analyzing the spectral weight maps (**Fig 4**) exploiting the distribution of weights alongside selected regions used as internal references of silicon and carbon contents. The scatter plots where each pixel is a datapoint relating the weights from different maps are presented in **Fig 7**.

The left column of **Fig 7** displays the scatter plots of the silicon K edge maps (1840–1880 eV) and the linear component in the 280–310 eV stacks. The observed proportionality, characterized by data points scattered along diagonal streaks, strengthens that the linear component in the 280–310 eV stacks primarily arises from the non-resonant X-ray absorption by the silica matrix.

Similarly, the graphs in the center column of **Fig 7** show the complementarity between the linear and the carbon K edge components in the 280–310 eV stacks. Here the data points form streaks with negative slopes, indicating the competition between the silica and the carbonaceous compounds for space within the lamellas. However, the slope of the streaks differs between the lamellas. Specifically, L1 and L4 exhibit strokes with steeper slopes than L2 and L3. Considering that L1 and L4 spectra is similar to lignin, whereas L2 and L3 are relatively closer to PGA or protein (**Fig 5**), differences in carbon densities (g_C_/cm^3^) (**S1 Table)** offer a plausible explanation for the observed differences in slope. A compound with higher carbon density (*e*.*g*., lignin) contributes relatively more carbon per unit of volume occupied within the silica matrix.

The next step in estimating the carbon content in phytoliths involves utilizing each data point in the center column of **Fig 7**, and comparing each data point to internal references that approximately represent the silica (zero carbon) and the carbonaceous (zero silica) components. The zero-carbon reference is calculated for each lamella and denoted by the black stars in the graphs of the center column of **Fig 7**. The horizontal position of the black star is approximately proportional to the mean thickness of the lamella. Subsequently, a normalized C signal is calculated as the carbon K edge weight of each pixel divided by the weight (black star) of the linear component calculated for each lamella. The results are presented as histograms in the right column of **Fig 7**. Pixels appearing below zero in the histogram are attributed to statistical noise, which appear as negative carbon signals in the scatter plot (center column of **Fig 7**) and reddish areas in the carbon maps (right column of **Fig 4**).

The reference point for zero-silica is located at the bottom-right corner of L1, where the characteristic silicon K edge feature is absent, and the carbon spectrum resembles lignin (**Fig 6**). This reference is represented by a vertical gray line across the histograms of **Fig 7**. Considering this reference as a 100% carbonaceous component, the calculated means across the distributions in the histograms are 9%, 17%, 25%, and 6% for L1, L2, L3, and L4, respectively. However, these percentages approximately represent volume fractions distributed between the 0% and 100% references. To estimate mass percentages, one can consider the ratio of the carbon density of lignin (0.87 g_C_/cm^3^) and the density of opal (2.1 g/cm^3^) (see **S1 Table**), which yields approximately 4%, 8%, 14%, and 3% (g_C_/g_opal_) for the lamellas L1, L2, L3, and L4, respectively. These percentages can then be compared to carbon contents in phytoliths determined using bulk methods, a comparison detailed in the subsequent discussion section.

## 4. Discussion

### 4.1. Location of carbon in phytoliths

The STXM analysis of the L1–L4 lamellas revealed significant variability in the carbon spatial distribution among the bilobate phytolith lamellas of the sugarcane stalks (**Fig 4**). Significant carbon variability among the phytoliths isolated from a common macroscopic sample was previously reported by Gallagher et al. [39], who used Raman spectroscopy to investigate sets of phytoliths isolated from *Sorghum bicolor* plants grown under variable conditions. In addition to demonstrating variability, Gallagher et al. [39] showed that carbon was distributed throughout the silica and not concentrated in the dark spots visible in light microscopy. The distribution of carbon throughout the silica was also observed by Alexandre et al. [35] using nanoSIMS. The carbon maps of **Fig 4** also confirmed the distributed carbon across the lamellas, but particularly abundant in L2 and L3, whereas carbon islands were predominant in L1 and L4. The occurrence of such carbon islands was not reported in the previous imaging of carbon in phytoliths, which could be attributed to artifacts arising from the wet chemical digestion performed to isolate the phytoliths [35, 39]. On the other hand, in the present study, the FIB sectioning of the phytolith eliminates the need for wet chemical digestion, and the presence of carbon islands surrounded by the silica matrix was evidenced as a minoritarian fraction of the phytolith carbon.

Another notable aspect of the carbon location is its complementarity with silicon distribution, as observed in **Fig 4** and expressed numerically in the scatter plots of **Fig 7**. This complementarity can be framed within broader carbon-silicon trade-offs in plants [49] and was already emphasized in the Hodson review [21], which considers that ‘*If the percentage carbon is high then percentage silicon must be low and vice versa*.’ The maps (**Fig 4**) and plots of map weights (**Fig 7**) bring the analysis of carbon-silicon complementarity into the realm of data-driven investigation with ≈100 nm spatial resolution. Noteworthy, the regions of the mixed silica-carbonaceous matrix were spatially extensive, spanning up to a few micrometers, a significant portion of the lamellas area. It is unclear how much of these silica-carbonaceous areas would endure wet chemical digestion protocols employed to isolate phytoliths for analysis.

### 4.2. Speciation of carbon in phytoliths

The primary spectral characteristics observed in the carbon target spectra constructed for L1–L4 (**Fig 5**) and raw spectra in selected ROIs (**Fig 6**) resembled the reference absorption spectrum derived from lignin (**Fig 5**). The main signature of this spectrum was the high relative intensity of the aromatic peak at ≈285.0 eV. However, it is important to note that this spectral feature does not conclusively indicate a macromolecular origin, thus it is more accurate to broadly attribute it to lignin-like aromatic compounds. In addition to lignin, the observations of carboxyl/carbonyl peaks at ≈288.5 eV provide evidence of a different type of chemical species. This ≈288.5 eV peak exhibited greater intensity in L3 (**Fig 5**), especially for the two ROIs located at the bottom of L3 (**Fig 6**). While the ≈288.5 eV peak could be potentially attributed to PGA (**Fig 5**), the attribution to protein was favored because it could account for both the aromatic (at lower relative intensity) and the carboxyl/carbonyl bands, as shown by the reference protein spectrum of **Fig 5**.

Observations of lignin-like compounds and proteins in phytoliths have precedents that are worth highlighting. Aromatic bands attributed to lignin were observed in part of the phytoliths analyzed by Gallagher et al. [39] using Raman spectromicroscopy. Moreover, the nanoSIMS analysis of phytoliths by Alexandre et al. [35] revealed that the carbon distributed in the silica matrix was associated with an N/C ratio consistent with amino acids. However, variability appears to be an inevitable characteristic when interpreting the chemical speciation of carbon in phytoliths. The spectral variability observed in larger sets of phytoliths analyzed by Raman spectromicroscopy [39, 40] is a testimony of the importance of variability for understanding carbon in phytoliths.

### 4.3. Quantity of carbon in phytoliths

The estimated carbon contents for lamellas L1, L2, L3, and L4 were 4%, 8%, 14%, and 3% (g_C_/g_opal_), respectively. These carbon percentages include various features within the field of view of the STXM image stacks, including carbon islands, carbon dispersed in the silica matrix, and carbon near the boundaries of the phytoliths, some of which may be extended beyond the silica matrix. These carbon percentages were of the same order of magnitude as those reported by Parr and Sullivan [14] for phytoliths from sugarcane leaves isolated with a microwave digestion method (4–20%), but notably higher than the observed values (0.1–0.5%) when a rapid digest method with H_2_SO_4_ and H_2_O_2_ was employed.

The variation in carbon percentages has been attributed to different types of phytolith carbon with varying susceptibilities to oxidation during wet chemical digestions. The lower percentages (≈0.1–0.5%) were attributed mainly to carbon well protected within lumen-type phytoliths, while the higher percentages (≈4–20%) also included carbon in cell wall type phytolith, which would be more susceptible to oxidation in wet-chemical digestion [14, 21, 50]. The extended regions of the mixed silicon-carbonaceous matrix observed in the weight maps (**Fig 4**) and scatter plots (**Fig 7**) adds complexity to this discussion. These regions contribute significantly to the carbon percentages reported in this study, but their susceptibility to wet chemical digestion remains unknown. This seems to be the main reason the observed carbon percentages are at the higher level (4–20%), whereas the lower level (≈0.1–0.5%) would be expected from the type of lumen phytolith here investigated. In broader terms, the dispersed type of carbon observed in **Fig 4** has an unknown level of connectivity and exposure to the external environment of the phytoliths, and thus, its susceptibility to wet-chemical digestion is unknown.

## 5. Conclusion

Phytoliths of biogenic silica produced by grasses are known to occlude organic carbon, with relevance to biosilica valorization in biorefineries, long-term carbon sequestration from the biosphere, and radiocarbon dating in archeology and paleontology. In previous studies, carbon in phytoliths has been analyzed after subjecting them to wet chemical protocols for isolation of the phytoliths. This study offered unprecedented experimental insights about the location, chemical speciation, and quantity of the carbon within phytoliths. The study employed an innovative method of phytolith sectioning into <500 nm thick lamella followed by the analysis of the carbon in phytoliths through synchrotron Scanning Transmission X-ray Microspectroscopy (STXM) with 100–200 nm pixel size. Our results obtained from four bilobate phytoliths from sugarcane stalk showed carbon fractions located as islands and dispersed in the opaline silica matrix. The most prevalent carbon spectral feature was an intense aromatic band attributed to lignin-like compounds, although regions consistent with proteinaceous carbon were also observed in the phytoliths. The calculated amounts of carbon from individual STXM image stacks (ranging from 3 to 14 wt%) were consistent with the carbon percentages already reported in bulk measurements using mild wet chemistry for phytolith isolation. Moreover, carbon islands that appeared completely surrounded by the silica matrix were found to be a minority, with the majority of the phytolith carbon found dispersed in the silica matrix, exhibiting a wide range of silica-carbon ratios and an unclear degree of exposure to the environment.

## Supporting information

S1 Fig Principal component analysis (PCA) of the image stacks in the 280–310 eV energy range. (PDF)

S2 Fig PC1 and PC2 eigenimages in the 280–310 eV energy range. (PDF)

S1 Table Comparison of approximate carbon densities calculated for different carbonaceous components. (PDF)

S1 Dataset Data of Figs 3, 5–7. (XLSX)
