# Supplementary material for: Location, speciation, and quantification of carbon in silica phytoliths using synchrotron scanning transmission X-ray microspectroscopy
Source: PLoS One. 2024 Apr 15;19(4):e0302009. doi: 10.1371/journal.pone.0302009 (PMC11018279; doi:10.1371/journal.pone.0302009)
Supplement: S1 Fig — (PDF) [file pone.0302009.s001.pdf]

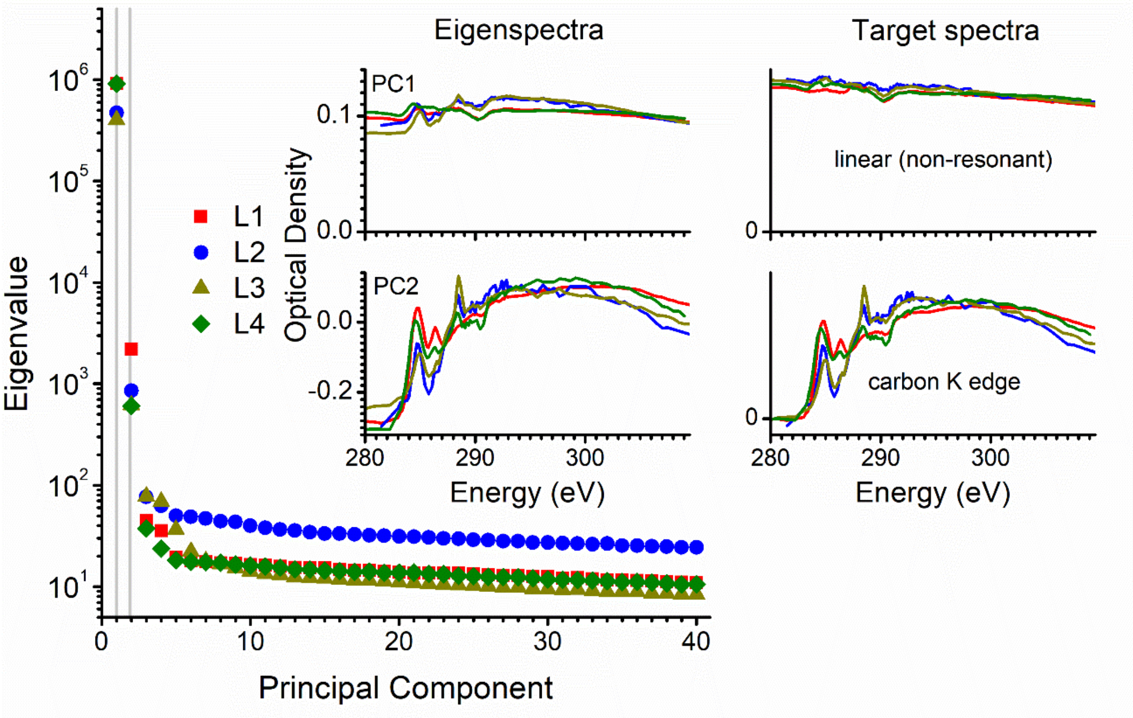

S1 Fig. Principal component analysis (PCA) of the image stacks in the 280–310 eV energy range. The scree plot indicates the significance of both PC1 and PC2 for each one of the four phytolith lamellas (L1–L4). Although PC3 and beyond may contain some statistical significance, only PC1 and PC2 are kept in the analysis, directing the interpretation of the spectral features that are undoubtedly significant. The insets at the left show the corresponding PC1 and PC2 eigenspectra. The insets at the right show the target spectra constructed as linear combinations of the PC1 and PC2 eigenspectra to represent (i) the linear (*i.e.*, non-resonant) and (ii) the carbon K edge spectral components. The similarity of the target spectra constructed for L1–L4 is remarkable, showing that a coherent interpretation of the measured spectra in the 280–310 eV range can be built as the combination of the linear and carbon target spectra.
