# Supplementary material for: Location, speciation, and quantification of carbon in silica phytoliths using synchrotron scanning transmission X-ray microspectroscopy
Source: PLoS One. 2024 Apr 15;19(4):e0302009. doi: 10.1371/journal.pone.0302009 (PMC11018279; doi:10.1371/journal.pone.0302009)
Supplement: S2 Fig — (PDF) [file pone.0302009.s002.pdf]

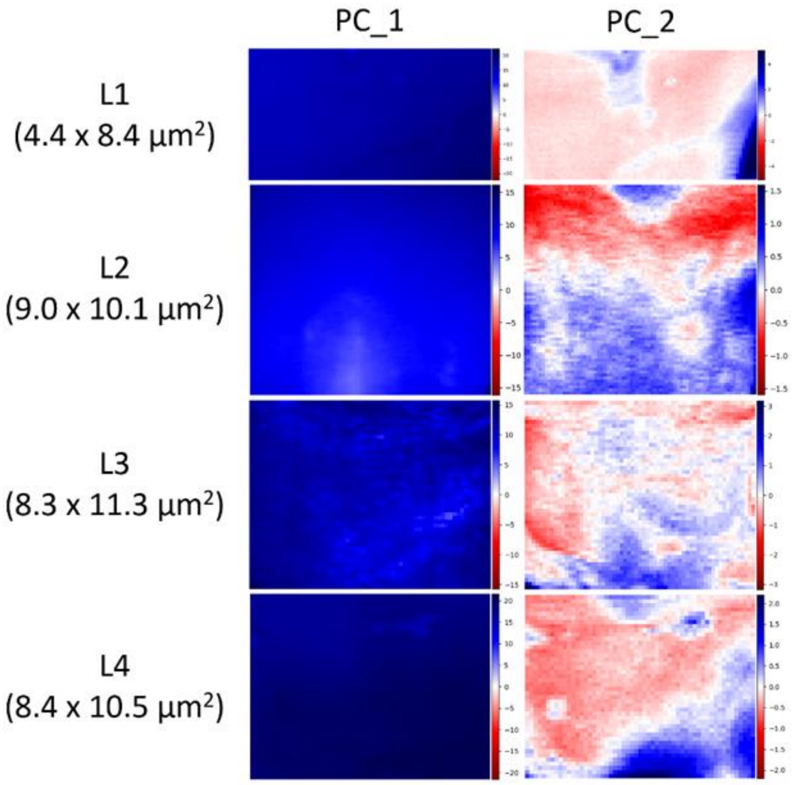

S2 Fig. PC1 and PC2 eigenimages in the 280–310 eV energy range. The eigenimages were obtained for each one of the phytolith lamellas (L1–L4).
