# Supplementary material for: Location, speciation, and quantification of carbon in silica phytoliths using synchrotron scanning transmission X-ray microspectroscopy
Source: PLoS One. 2024 Apr 15;19(4):e0302009. doi: 10.1371/journal.pone.0302009 (PMC11018279; doi:10.1371/journal.pone.0302009)
Supplement: S1 Table — (PDF) [file pone.0302009.s003.pdf]

S1 Table. Comparison of approximate carbon densities calculated for different carbonaceous components. The carbon densities are to be compared with an opal density of ≈2.1 g/cm^3 [1].

Component	Density (g/cm^3)	Carbon wt.%	Carbon density (g_C/cm^3)
**Lignin**	1.3 [2]	67 [4]	0.87
**Protein**	1.4 [3]	53 [5]	0.74
**Polygalacturonic acid**	1.5 [2]	41 [4]	0.61

References:

[1] http://webmineral.com/data/Opal

[2] Karunakaran C, Christensen CR, Gaillard C, et al (2015) Introduction of Soft X-Ray Spectromicroscopy as an Advanced Technique for Plant Biopolymers Research. PLoS One 10:e0122959.

[3] Fischer H, Polikarpov I, Craievich AF (2009) Average protein density is a molecular-weight-dependent function. Protein Sci 13:2825–2828.

[4] Calculated from molecular formulas. For lignin, the precursor coniferyl alcohol was considered.

[5] Rouwenhorst RJ, Frank Jzn J, Scheffers WA, van Dijken JP (1991) Determination of protein concentration by total organic carbon analysis. J Biochem Biophys Methods 22:119–128.
